# Acute Disseminated Encephalomyelitis Related to a Cytomegalovirus Infection in an Immunocompetent Patient

**DOI:** 10.7759/cureus.12795

**Published:** 2021-01-19

**Authors:** Rafael C Da Silva, Guilherme B Aguiar, Carolina Kamer, Lais Farias, Josie Matsuda

**Affiliations:** 1 Internal Medicine, Piedmont Athens Regional Medical Center, Athens, USA; 2 Neurological Surgery, Santa Casa De Sao Paulo, Sao Paulo, BRA; 3 Medicine, School of Medicine, Universidade para o Desenvolvimento do Alto, Rio do Sul, BRA

**Keywords:** cytomegalovirus (cmv), acute disseminated encephalomyelitis, case report, immunocompetent, steroids, systemic steroids, myelin, white matter

## Abstract

Cytomegalovirus (CMV) infection can cause acute disseminated encephalomyelitis (ADEM). However, it is rare in immunocompetent people. We describe a 17-year-old patient who was brought with flu-like symptoms. After one week, she experienced rapidly progressive weakness in all four extremities, followed by coma. The neurologic examination showed no response to verbal and pain stimuli. A Babinski sign was noted in both lower extremities, along with clonus and hyperreflexia in all four limbs. Brain magnetic resonance imaging (MRI) demonstrated extensive areas of hyperintense signal on fluid-attenuated inversion recovery (FLAIR) sequences in the white matter which was asymmetrically distributed in both hemispheres, as well as in the brainstem and cerebellar peduncles, compatible with acute demyelinating lesions. Cerebrospinal fluid (CSF) showed mild lymphocytic pleocytosis and normal glucose levels. Polymerase chain reaction to herpes simplex virus was negative. Serum immunoglobulin G (IgG) and immunoglobulin M (IgM) were positive for cytomegalovirus. The patient was treated with methylprednisolone pulse therapy for five days. Subsequently, the patient showed neurologic improvement with the recovery of consciousness and muscle strength. In terms of prognosis, in patients with ADEM, the sooner the diagnosis, the better the outcome. The cornerstone of treatment is immunosuppression with steroids. Some patients require intravenous immunoglobulin G (IVIG) or plasmapheresis, and in refractory cases, cyclophosphamide is used.

## Introduction

Acute disseminated encephalomyelitis (ADEM) is an inflammatory demyelinating disease of the central nervous system [1-2]. It is characterized by a monophasic clinical course. Extensive white matter lesions are generally found on brain magnetic resonance imaging (MRI). ADEM can also be further characterized etiologically into three types: post-infectious, post-vaccination, and idiopathic [2]. ADEM related to the cytomegalovirus (CMV) infection is a rare condition in immunocompetent people with only a few cases reported [2-4]. Therefore, we report a case of a young patient with a severe form of ADEM following a CMV infection with a favorable outcome.

## Case presentation

A 17-year-old female presented with a history of flu-like symptoms (low-grade fever and body aches). One week after the initial presentation, she experienced a rapidly progressive and symmetric weakness in all four extremities. There was no visual or sensory impairment. No abdominal, respiratory, or cardiovascular symptoms were reported. The past medical history was negative, and the patient had no previous surgery. There was no current medication or recent vaccination history. She was not sexually active, and there was no environmental or sickly contact exposure. Subsequently, the patient became lethargic and progressed to a coma. She did not have seizures. On admission, the neurologic examination showed no response to verbal and pain stimuli. There was no neck rigidity. Kernig's sign was negative. A Babinski's sign was noticed in both extremities, along with clonus. Hyperreflexia was elicited in all four limbs. There was a maculopapular rash all over the trunk and extremities. 

A list of differentials were considered, which included infective meningitis, subarachnoid hemorrhage, encephalitis, multiple sclerosis, neuromyelitis optica (NMO), central nervous system (CNS) vasculitis, brain tumor, acute intoxication, and metabolic/endocrinologic conditions. A complete blood count and basic metabolic panels were normal. Urine toxicologic screening was negative. Head computed tomography (CT) showed no abnormality, and cerebrospinal fluid (CSF) analysis was unremarkable. Herpes virus, human immunodeficiency virus (HIV), CMV, rubeola, measles, and hepatitis serology panels were pending. NMO-immunoglobulin G (IgG) and myelin oligodendrocyte glycoprotein (MOG) antibodies were not done due to technical conditions. 

**Figure 1:**
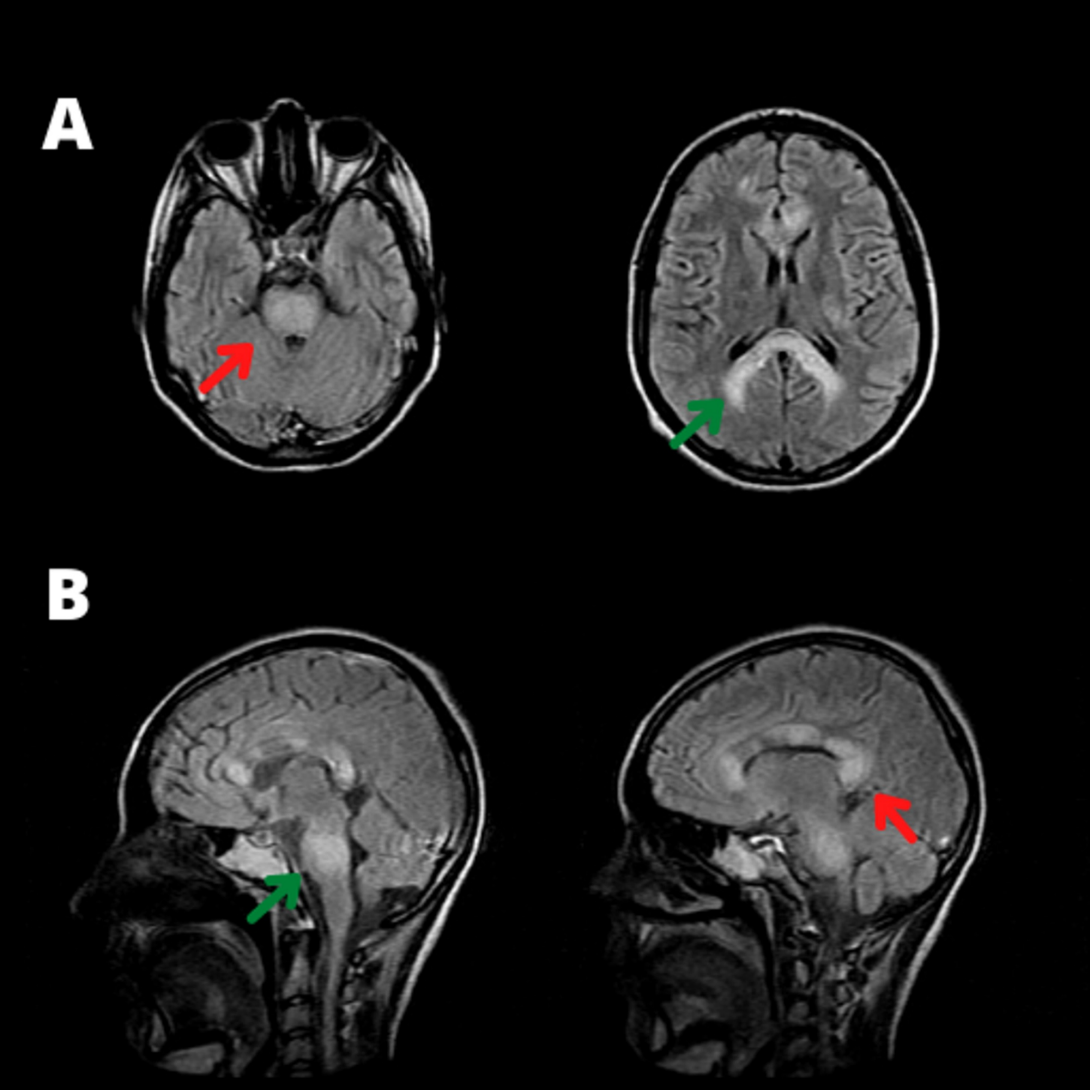
FLAIR sequences of axial (A) and sagittal (B) planes demonstrate the usual pattern of demyelinating lesions of the white matter with a predominance of lesions in the brainstem (green arrows), as well as in the posterior aspect of the corpus callosum and internal capsule (red arrows). FLAIR: fluid attenuation inversion recovery

She was put on acyclovir, ceftriaxone, and vancomycin to cover both bacterial meningitis and herpetic encephalitis. Subsequently, HIV and serologies for hepatitis, rubella, and measles were negative. Polymerase chain reaction (PCR) for herpes virus in the CSF was negative as well. However, serum IgG and immunoglobulin M (IgM) for CMV demonstrated high titles, confirming recent systemic infection. A lumbar puncture was repeated, and at this time, the CSF showed a severe hypercellularity of 426 white cells/μL (normal range: 0 - 5 cells/μL) with 65% lymphocytes and 62 mg/dL of protein (normal range: 15 - 60 mg/dL). A glucose level was 95 mg/dL (normal range: 50 - 80 mg/dL). CSF oligoclonal bands (OCB) were not detected. MRI of the brain demonstrated multifocal, poorly marginated, and extensive areas of hyperintense signal on fluid-attenuated inversion recovery (FLAIR) sequences in the white matter, asymmetrically distributed in both hemispheres, as well as in the brainstem and cerebellar peduncles, compatible with acute demyelination lesions (Figure 1). Cervical and thoracic spine MRI scans did not demonstrate an altered signal. 

At this point, acute disseminated encephalomyelitis (ADEM) was diagnosed, and pulse therapy with methylprednisolone (15 mg/kg/day) was initiated for five days. On the third day of treatment, the patient showed neurologic improvement with the recovery of consciousness. She was able to move all four limbs and muscle power became normal. She had progressive neurologic recovery and was discharged after seven days with no motor deficits and mild cognitive decline.

## Discussion

The clinical picture, laboratory, and imaging studies of this patient were compatible with ADEM following a CMV infection. In immunocompetent hosts, primary infection with CMV is usually self-limited and sometimes asymptomatic. The diagnosis of acute infection was based on flu-like prodromes, a diffuse cutaneous rash, and high titles of serum IgG and IgM antibodies. ADEM, in general, is preceded either by a viral infection affecting the respiratory system or after vaccination [1-2]. According to the International Pediatric Multiple Sclerosis Group [5], our patient met the criteria for ADEM based on an acute first clinical demyelinating event accompanied by encephalopathy and neuroimaging showing multifocal asymmetrical lesions in the white matter. 

Sometimes, ADEM and acute infectious encephalitis can be hard to distinguish. Some clinical features can be used, such as a history of fever and acute encephalopathy. The laboratory profile can be supportive to differentiate between these two conditions [5-6]. The characteristic picture of ADEM is a young patient presenting with sudden onset of neurological multifocal and progressive signs affecting the central nervous system [5-6]. Also, ADEM sometimes can be more restricted and present with transverse myelitis. Multiple sclerosis can present as the first episode of ADEM [4, 7]. On the other hand, infectious encephalitis is characterized by the sudden onset of headache, confusion, meningism, aphasia or mutism, and deep coma [2, 6]. Seizures occur in 61% of cases during the course of the illness [7]. In that sense, herpes simplex virus type 1 (HSV-1) accounts for the majority of cases of encephalitis [6]. 

In terms of pathogenesis, ADEM is an autoimmune disorder of the CNS, triggered by an environmental stimulus in susceptible individuals [8-9]. Molecular sequencing of viral or bacterial microorganisms may share antigenic properties with myelin. This molecular mimicry can activate T-cells and provoke inflammation, followed by demyelination [9-10]. One of the autoantibodies detected in the CSF of patients with ADEM is serum IgG antibodies to myelin oligodendrocyte glycoprotein (MOG) [10]. This IgG antibody is usually found in seronegative neuromyelitis optica spectrum disorder (NMOSD). Also, it was demonstrated that MOG-positive ADEM patients were more likely to have large, bilateral, and widespread lesions on MRI. However, they were more likely to show a more favorable clinical outcome [7]. Due to technical issues, our patient did not have MOG antibodies measured which, in practical terms, did not determine management. 

In terms of evaluation and diagnostic studies, brain and spinal MRI scans are the modalities of choice [1, 6, 11-12]. Hyperintense lesions on T2-weighted images, as well as on FLAIR sequences, are expected, as shown in this case. However, it is important to note that ADEM may present initially with normal brain and spinal MRIs and repeated imaging shall be warranted [3]. The value of a head CT scan is to rule out life-threatening events, such as subarachnoid hemorrhages or a massive stroke; frequently, it is unremarkable. The CSF might reveal lymphocytic pleocytosis and slightly elevated protein in approximately 50% to 80% of cases [1-2, 8]. 

When considering treatment, the mainstay is immunosuppression with high-dose steroids [1-2, 4-5]. However, acyclovir and broad-spectrum antibiotics must be considered empirically until further evaluation to rule out herpes encephalitis and bacterial infection in face of its severity [5-6, 8]. In cases of non-responsiveness to initial treatment with steroids, IVIG [13], plasmapheresis [14-15], or even cyclophosphamide might be considered [3].

## Conclusions

Acute disseminated encephalomyelitis (ADEM) is an acute demyelinating disease, mostly with a monophasic course. It manifests with a broad spectrum of presentation and with characteristic findings on MRI. However, in some cases, the MRI brain and spinal could be normal during the initial course. ADEM is associated with CMV infection; however, it is rare in immunocompetent individuals. The sooner the diagnosis, the better the outcomes. The cornerstone of therapy is steroids and some patients will require IVIG or plasmapheresis. In refractory cases, cyclophosphamide is used. 
